# Assembly of Complete Genome Sequences of Negative-Control and Experimental Strain Variants of Staphylococcus aureus ATCC BAA-39 Selected under the Effect of the Drug FS-1, Which Induces Antibiotic Resistance Reversion

**DOI:** 10.1128/MRA.00579-19

**Published:** 2019-07-25

**Authors:** Monique Joubert, Oleg N. Reva, Ilya S. Korotetskiy, Sergey V. Shvidko, Sergey V. Shilov, Ardak B. Jumagaziyeva, Sabina T. Kenesheva, Natalya A. Suldina, Aleksandr I. Ilin

**Affiliations:** a Center for Bioinformatics and Computational Biology (CBCB), Department of Biochemistry, Genetics and Microbiology, University of Pretoria, Pretoria, South Africa; b Scientific Center for Anti-Infectious Drugs (SCAID), Almaty, Kazakhstan; University of Rochester School of Medicine and Dentistry

## Abstract

Staphylococcus aureus ATCC BAA-39 is the reference organism for a multidrug-resistant Staphylococcus aureus (MRSA) strain that was used to study drug-induced resistance reversion by an iodine-containing nanomolecular complex, FS-1. PacBio sequencing was performed on both the experimental and control strains, followed by genome assembly, variant calling, and DNA modification profiling.

## ANNOUNCEMENT

The iodine-containing nanomolecular complex FS-1 induces a reversion of drug-resistant bacteria into sensitive phenotypes (1). FS-1 was designed to supplement antibiotic treatment therapy against drug-resistant tuberculosis (2) and has been shown to be successful against multidrug-resistant Staphylococcus aureus (MRSA) (3). To investigate the molecular mechanisms of this phenomenon, Staphylococcus aureus ATCC BAA-39 was used as a model organism. The strain was cultivated with a sublethal concentration of FS-1 (12 μg/ml) in 10 daily passages. In parallel, the culture was cultivated in the same medium without FS-1 to serve as a negative control (NC). Afterwards, chromosomal DNA samples were prepared in 3 repeats. DNA samples were extracted from bacterial cells using the PureLink genomic DNA kit (Thermo Fisher). The samples were sequenced by Macrogen (South Korea) on single-molecule real-time (SMRT) 8Pac V3 cells following the SMRTbell 20-kb protocol. The average *N*_50_ value and the average length of the subreads were 15 kb and 10 kb, respectively.

S. aureus ATCC BAA-39 (4) was isolated in 2010 from a nasal clinical sample (BioProject accession number PRJNA50533). The whole-genome assembly comprising 83 contigs was published in 2010 (GCA_000146385). The PacBio reads generated in this research have provided genome coverages of 966× and 906× for the FS and NC variants, respectively. PacBio subreads were published at the NCBI under BioProject accession number PRJNA480363. The complete genome assembly of the PacBio reads was performed using SMRT Link v5.0.1 pipelines (https://www.pacb.com/support/software-downloads/) with default parameters. The *de novo* assembly pipeline makes use of Hierarchical Genome Assembly Process (HGAP v4), with a default minimum seed length of 6 kb. Two genome variants, NC and FS, were obtained as single-contig sequences without gaps and ambiguities. The completeness of the final assemblies was evaluated using the Benchmarking Universal Single-Copy Orthologs (BUSCO v3.0.2) software (5) with parameters set to default. The lengths of the NC and FS genomes were 2,791,218 bp and 2,792,888 bp, respectively, with an average GC content of 32.9%. Genomes were annotated using the Rapid Annotations using Subsystems Technology (RAST) server (6). A methicillin resistance staphylococcal casette chromosome *mec* element (SCC*mec*) containing the gene *mecA,* which encodes the penicillin-binding protein 2a (7, 8), was found in both genomes. Epigenetic modifications of nucleotides were detected in the genomes by aligning PacBio reads against the complete genome sequences, followed by base call kinetic analysis using the SMRT Link v5.0.1 software package. Identified *N*^6^-adenosine methylation motifs were published at the NCBI together with the complete genome sequences. The obtained genomes will be used in upcoming experiments to study the mechanisms of antibiotic resistance reversion induced by FS-1 in S. aureus. A working hypothesis is that FS-1 may cause inactivation of the drug resistance determinants in SCC*mec* by an increased rate of mutations, gene regulation alteration, or epigenetic modifications.

### Data availability.

The genome sequences are available from the NCBI under accession numbers CP033505 and CP033506 (BioProject accession number PRJNA480363) for the NC and FS variants, respectively. The SRA accession numbers are SRR9077059 for NC and SRR9067444 for FS.

## References

[1] Ilin AI, Kulmanov ME, Korotetskiy IS, Islamov RA, Akhmetova GK, Lankina MV, Reva ON 2017 Genomic insight into mechanisms of reversion of antibiotic resistance in multidrug resistant *Mycobacterium tuberculosis* induced by a nanomolecular iodine-containing complex FS-1. Front Cell Infect Microbiol 7:151. doi:10.3389/fcimb.2017.00151. 28534009 PMC5420568

[2] Kalykova A, Kustova T, Sakipova Z, Ibragimova N, Islamov R, Vetchý D, Ilin A 2016 Acute and subchronic toxicity studies of the original drug FS-1. Acta Vet Brno 85:9–16. doi:10.2754/avb201685010009.

[3] Korotetskiy I, Shilov S, Shvidko S, Jumagaziyeva A, Suldina NA, Korotetskaya NV, Ilin A, Reva O 2017 Transcriptional response of the multidrug resistant *Staphylococcus aureus* following FS-1 exposure. Eurasian J Appl Biotechnol 2017:43–48. doi:10.11134/btp.3.2017.6.

[4] Garrigós C, Murillo O, Lora-Tamayo J, Verdaguer R, Tubau F, Cabellos C, Cabo J, Ariza J 2012 Efficacy of daptomycin-cloxacillin combination in experimental foreign-body infection due to methicillin-resistant *Staphylococcus aureus*. Antimicrob Agents Chemother 56:3806–3811. doi:10.1128/AAC.00127-12. 22585211 PMC3393403

[5] Simao FA, Waterhouse RM, Ioannidis P, Kriventseva EV, Zdobnov EM 2015 BUSCO: assessing genome assembly and annotation completeness with single-copy orthologs. Bioinformatics 31:3210–3212. doi:10.1093/bioinformatics/btv351. 26059717

[6] Aziz RK, Bartels D, Best AA, DeJongh M, Disz T, Edwards RA, Formsma K, Gerdes S, Glass EM, Kubal M, Meyer F, Olsen GJ, Olson R, Osterman AL, Overbeek RA, McNeil LK, Paarmann D, Paczian T, Parrello B, Pusch GD, Reich C, Stevens R, Vassieva O, Vonstein V, Wilke A, Zagnitko O 2008 The RAST server: Rapid Annotations using Subsystems Technology. BMC Genomics 9:75. doi:10.1186/1471-2164-9-75. 18261238 PMC2265698

[7] Holden MT, Feil EJ, Lindsay JA, Peacock SJ, Day NP, Enright MC, Foster TJ, Moore CE, Hurst L, Atkin R, Barron A, Bason N, Bentley SD, Chillingworth C, Chillingworth T, Churcher C, Clark L, Corton C, Cronin A, Doggett J, Dowd L, Feltwell T, Hance Z, Harris B, Hauser H, Holroyd S, Jagels K, James KD, Lennard N, Line A, Mayes R, Moule S, Mungall K, Ormond D, Quail MA, Rabbinowitsch E, Rutherford K, Sanders M, Sharp S, Simmonds M, Stevens K, Whitehead S, Barrell BG, Spratt BG, Parkhill J 2004 Complete genomes of two clinical *Staphylococcus aureus* strains: evidence for the rapid evolution of virulence and drug resistance. Proc Natl Acad Sci U S A 101:9786–9791. doi:10.1073/pnas.0402521101. 15213324 PMC470752

[8] Jani M, Sengupta S, Hu K, Azad RK 2017 Deciphering pathogenicity and antibiotic resistance islands in methicillin-resistant *Staphylococcus aureus* genomes. Open Biol 7:170094. doi:10.1098/rsob.170094. 29263245 PMC5746543

